# Comprehensive Review of Dietary Probiotics in Reducing Aflatoxin B_1_ Toxicity

**DOI:** 10.3390/toxins17100482

**Published:** 2025-09-26

**Authors:** Dasol Choi, Xingrui Fan, Jae-Hyuk Yu

**Affiliations:** 1 Department of Materials Science & Engineering, University of California, Los Angeles, CA 90095, USA; dasolchoi30@ucla.edu; 2 Department of Food Science, University of Wisconsin-Madison, Madison, WI 53706, USA; xfan73@wisc.edu; 3 Food Research Institute, University of Wisconsin-Madison, Madison, WI 53706, USA; 4 Department of Bacteriology, University of Wisconsin-Madison, Madison, WI 53706, USA

**Keywords:** aflatoxins, probiotic lactic acid bacteria (LAB), anti-carcinogenic effect, hepatocellular carcinoma, bio-adsorbent

## Abstract

Aflatoxin B_1_ (AFB_1_), the most potent and widespread mycotoxin produced by *Aspergillus flavus* and *Aspergillus parasiticus*, poses a significant global threat to food safety and human health, with chronic exposure strongly linked to hepatocellular carcinoma (HCC). While physical and chemical detoxification approaches exist, their limitations have led to an increased interest in biological strategies, particularly probiotic interventions. In this review, we synthesize current in vivo and clinical evidence on the ability of probiotic lactic acid bacteria—including *Lactobacillus casei* Shirota, *Lactobacillus rhamnosus* GG, *Lactobacillus rhamnosus* LC705, *Lactococcus lactis*, and selected *Bifidobacterium* species—to reduce AFB_1_ absorption and toxicity. We summarize mechanistic insights into cell wall adsorption, gut microbiota modulation, intestinal barrier protection, and antioxidant enhancement. Clinical trials have shown reductions in AFB_1_ biomarkers following probiotic supplementation, supporting their translational potential for human health. However, clinical evidence remains limited by small sample sizes, short intervention periods, and variability in endpoints. Collectively, this review consolidates mechanistic, preclinical, and clinical findings to position probiotic lactic acid bacteria as promising biological countermeasures against AFB1-induced hepatocellular carcinoma.

## 1. Introduction

Filamentous fungi, including *Aspergillus*, *Penicillium*, and *Fusarium*, are prevalent in soils, farms, and other agricultural environments. Many species produce mycotoxins, which were defined by Bennett [1] as “natural products produced by fungi that evoke a toxic response when introduced in low concentration to higher vertebrates and other animals by a natural route.” As low-molecular-weight bioactive molecules, mycotoxins frequently infiltrate human food systems and animal feed supplies, establishing themselves as persistent threats to global health security [2]. The magnitude of mycotoxin contamination across global food systems represents a substantial crisis. Earlier estimates by the United Nations Food and Agriculture Organization (UN FAO) and World Health Organization (WHO) placed global mycotoxin contamination in crops at around 25%, but more recent assessment by Eskola et al. [3] suggests that the actual prevalence is considerably higher across food crops worldwide [3,4]. While the scientific literature documents nearly 400 distinct mycotoxin compounds, only a select group demonstrates the combination of extreme toxicity, environmental persistence, and widespread distribution that creates significant public health concerns. This critical subset encompasses aflatoxins (AF), ochratoxins (OT), zearalenone (ZEA), T-2 toxin, fumonisins (FB), deoxynivalenol (DON), and related trichothecene compounds [5]. The inherent chemical and thermal stability of these molecules renders conventional food processing techniques largely ineffective for their elimination [6,7].

**Figure 1:**
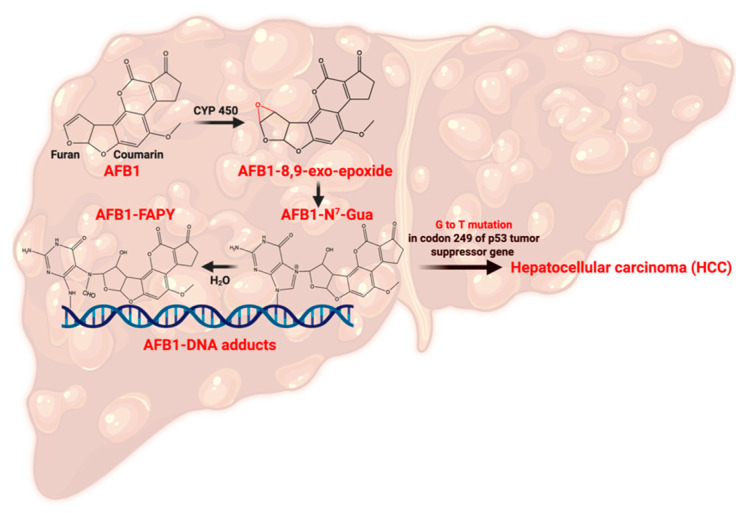
Pathway of AFB_1_ metabolism leading to hepatocellular carcinoma (HCC). Created with Biorender.com.

The discovery of AF emerged from tragedy when an enigmatic illness, subsequently termed “Turkey X disease,” devastated British poultry in 1960, claiming approximately 100,000 turkey lives [8]. AF-producing fungi, including *Aspergillus flavus* and *Aspergillus parasiticus*, are common in crops including maize, corn, wheat, and sorghum [9,10,11,12]. Contemporary risk assessments indicate that between 4.5 and 5.5 billion individuals globally face ongoing AF exposure through dietary consumption [10,13]. Within the AF family, aflatoxin B_1_ (AFB_1_) stands as the most biologically potent member, capable of inducing genotoxicity, oncogenesis, and immunological dysfunction [14,15]. Therefore, the U.S. Food and Drug Administration (FDA) imposes an action level of 20 parts per billion (ppb) for total AF in foods. The carcinogenic potential of AFB_1_ has earned it a classification as a Group I carcinogen by the International Agency for Research on Cancer (IARC), with hepatic malignancy representing its primary oncological consequence [16,17]. Upon consumption, AFB_1_ undergoes hepatic biotransformation through cytochrome P450 enzymatic pathways, specifically involving CYP1A2 and CYP3A4 isoforms, resulting in the formation of aflatoxin-8,9-exo-epoxide, a highly reactive intermediate [17,18,19]. This metabolic intermediate demonstrates high affinity with DNA, RNA, and protein molecules [20,21]. The predominant DNA lesion involves adduct formation at guanine N^7^ positions, which is recognized as a biomarker for hepatocarcinogenic risk assessment [19,22]. These DNA modifications frequently compromise p53 tumor suppressor function through characteristic G→T transversion mutations occurring at codon 249, establishing conditions conducive to hepatocellular carcinoma (HCC) development (see Figure 1) [11,20,23,24,25]. Numerous studies have conclusively established chronic AF exposure as a principal etiological factor in HCC development [26,27,28,29,30,31]. Beyond direct genotoxic effects, aflatoxicosis initiates inflammation by damaging the liver tissue and results in elevated levels of apoptosis and carcinogenic activities [32,33,34].

HCC represents a central global malignancy, ranking fifth among all cancer types and third in cancer-associated mortality worldwide [35]. Current estimates attribute 4.6–28.2% of the global liver cancer burden directly to AF exposure [36,37]. Therefore, scientists are actively looking for strategies to mitigate the impact of AF on human health. In recent years, the human gut microbiome and probiotics have been extensively studied for potential anticarcinogenic functions, and interest has been raised in modifying the gut microbiome to reduce AFB_1_ bioavailability and toxicity [38]. The relationship between gut microbiota and AFB_1_ uptake has attracted considerable scientific attention and has been widely investigated. This review provides a critical synthesis of mechanistic, preclinical, and clinical data on probiotic interventions for AFB_1_ toxicity. It specifically evaluates the molecular mechanisms of action, summarizes key findings from animal models and human intervention studies, and discusses both the therapeutic potential and current limitations of probiotic strategies for AFB_1_ detoxification.

## 2. AFB_1_-Induced Alterations of Gut Microbiota

### 2.1. Adsorption of AFB_1_ in the GI Tract

The gastrointestinal tract (GI) serves as a crucial barrier, offering multifaceted defense mechanisms against pathogenic microorganisms, environmental toxins, and harmful xenobiotics. However, exposure to AFB_1_ disrupts this protective interface. Studies demonstrate that AFB_1_ exposure leads to a dose-dependent increase in intestinal crypt depth, even when villus length remains unchanged, indicating structural alterations that undermine normal mucosal function. These morphological changes, combined with reductions in intestinal weight that impaired nutrient absorption and diminished mucosal health, facilitated greater susceptibility to toxins like AFB_1_ [39,40]. Moreover, in vivo studies have shown that AFB_1_ exposure increases the plasma lactulose-to-rhamnose ratio, a marker of intestinal permeability since lactulose absorption is enhanced when tight junction integrity is compromised [41]. This disruption is accompanied by leukocyte and lymphocyte infiltration into the lamina propria [2], suggesting acute inflammation and impaired gut–liver axis function. The cytotoxic effects of AFB_1_ are primarily attributed to the excessive generation of intracellular reactive oxygen species (ROS), which triggers the release of high levels of lactate dehydrogenase (LDH) and consequently damages cell membranes and DNA integrity [42]. Within the GI tract, AFB_1_ has been linked to intestinal barrier disruption, altered cell proliferation, and increased apoptosis [11]. While detoxification of AFB_1_ primarily occurs in the large intestine through microbial biotransformation into less toxic derivatives, more than 80% of the toxin is rapidly absorbed in the duodenum by passive diffusion [11,42]. Therefore, restoration of barrier function and protection against these adverse effects can be achieved by modulating the gut microbiota, highlighting its critical role in maintaining intestinal health.

### 2.2. Modulation of Gut-Health-Induced Microbiota

Exposure to AFB_1_ induces significant changes in gut microbiota composition, and how microbiota reacts to AFB_1_ depends on the exposure level, as described in Table 1. A consistent observation across multiple studies is that AFB_1_ exposure modulates gut microbiota composition, characterized by a reduction in *Bacteroidetes* and a concomitant increase in *Firmicutes* [43,44,45,46]. These two phyla represent the dominant constituents of the gut microbiome, and their imbalance reflects a significant shift in microbial homeostasis [44]. This shift suggests that members of *Firmicutes* may possess greater tolerance to AFB_1_, enabling them to outcompete other taxa under toxin stress. *Firmicutes* comprise several Gram-positive genera, including *Lactobacillus* and *Streptococcus*, which belong to the LAB group. LAB can remove AFB_1_ through binding to cell wall structures [47,48]. Nevertheless, the impact of AFB_1_ on specific *Firmicutes* taxa remains inconsistent across studies. For instance, *Streptococcus* spp. and *Lactococcus* spp. showed pronounced declines at AFB_1_ concentrations ranging from 5 to 75 ppb [49], and *Lactobacillus* spp. abundance decreased by 50.5% (*p* < 0.05) in piglets when exposed to 320 ppb of AFB_1_ [50]. Conversely, other investigations have reported increased total LAB populations at 1500–2000 ppb AFB_1_ (*p* < 0.05), while a significant reduction was observed in broilers exposed to 1000 ppb [51]. Such discrepancies suggest that LAB responses to AFB_1_ are highly dose-dependent and possibly influenced by host species and diet. In addition to compositional changes, exposure to AFB_1_ affects microbial metabolism. At 2500 ppb AFB_1_, reductions in short-chain fatty acids (SCFAs) were observed, alongside depletion of SCFA-producing LAB strains [3,52]. This decline in SCFAs, critical for maintaining intestinal homeostasis, highlights the potential for AFB_1_ to disrupt gut metabolic activity. Interestingly, some taxa appear to proliferate under high toxin exposure, such as *Bifidobacterium* spp. abundance increased significantly at 10,000 ppb AFB_1_ (*p* = 0.001), accompanied by elevated xylanase (*p* = 0.005) and cellulase (*p* = 0.002) activities, suggesting an enzymatic adaptation to counter intestinal microecological imbalance [53]. Collectively, these findings indicate that AFB_1_ exerts profound, dependent effects on gut microbiota composition, toxin’s exposure level and metabolism. Shifts in the LAB populations highlight the dynamic interactions between probiotics and AFB_1_ stress, offering new insights into the role of beneficial microbes in AFB_1_ detoxification.

## 3. Methods for Detoxifying AFB_1_ in the GI Tract

A variety of physical, chemical, and biological approaches have been explored to inactivate and detoxify AFB_1_ in food and feed [54,55,56,57]. For example, clay minerals are widely used to selectively or nonspecifically adsorb mycotoxins in the GI tract [55,58]. Inorganic adsorbents, such as aluminosilicates, have also demonstrated a strong binding capacity for AFB_1_ in animal studies [59,60,61,62,63]. While they are generally recognized as safe (GRAS) for dietary inclusion, no adsorbent has yet been approved by the U.S. Food and Drug Administration (FDA) for clinical treatment of aflatoxicosis. Moreover, these strategies are often costly and impractical for widespread application. Despite the benefits of existing physical and chemical methods, there remains an urgent need for more effective, safe, and affordable detoxification strategies. In recent years, biological processes, particularly probiotic interventions, have gained increasing attention as promising alternatives. These approaches utilize the natural binding and detoxifying capacities of the gut microbiota to mitigate AFB_1_ toxicity in the gastrointestinal tract.

### Probiotics LAB as Potential Detoxifiers of AFB_1_

According to the World Health Organization (WHO), probiotics are defined as “live microorganisms that, when administered in adequate amounts, provide a health benefit to the host” [64]. Beyond their well-recognized role in gut health and microbiota restoration, probiotics have also been investigated for their ability to detoxify AF in food and the GI tract. Some strains are capable of modifying the chemical structure of AFB_1_, converting it into less toxic or non-toxic metabolites. However, these transformations do not always eliminate toxicity entirely, as certain products, such as aflatoxicol, may retain harmful effects [65,66]. An alternative mechanism involves direct binding of AFB_1_ to microbial cells, which reduces its intestinal absorption and subsequent systemic toxicity. Among probiotics, LAB have shown particularly strong binding affinities toward AFB_1_ [48,67,68,69]. Both viable and non-viable LAB cells can effectively adsorb AFB_1_, indicating that the detoxification mechanism is linked to cellular structural components rather than active metabolism [70]. Supporting this, Haskard et al. [71] demonstrated that periodate treatment significantly reduced the AFB_1_-binding capacity of *Lactobacillus rhamnosus* GG, implicating carbohydrate structures in the cell wall as the key binding sites. In contrast, treatments with proteases or lipases exerted minimal effects, suggesting that proteins and lipids play a negligible role in AFB_1_ adsorption [70]. LAB are Gram-positive bacteria characterized by a thick peptidoglycan cell wall, a carbohydrate-rich structure regarded as the principal component responsible for sequestering AF. This was confirmed by Lahtinen et al. [72], who found that cell wall extracts of *L. rhamnosus* GG retained a binding capacity of 81% for AFB_1_, comparable to that of intact viable cells (84%), whereas purified exopolysaccharides bound less than 1%. Similarly, Zhu et al. [73] reported that highly purified (97.75%) peptidoglycan isolated from *Limosilactobacillus reuteri* adsorbed 64.3–75.9% of AFB_1_ in vitro. These findings indicate that peptidoglycan and related polysaccharide structures are the primary contributors to AFB_1_ adsorption onto LAB. The formation of AFB_1_–LAB complexes prevents toxin absorption via paracellular diffusion, thereby reducing the risk of hepatocarcinogenesis. Among probiotic LAB, *Lactobacillus*, *Bifidobacterium*, and *Lactococcus* are the most widely studied, with reported AFB_1_-binding capacities ranging from 5.6% to 59.7% [48]. In particular, *Lactobacillus* spp. consistently demonstrate high efficacy in sequestering AF, highlighting their potential as promising biological strategies for AFB_1_ mitigation [68,69,71].

Not all LAB strains exhibit the same capacity to bind AFB_1_. In a screening of 20 LAB strains conducted by Peltonen, el-Nezami, Haskard, Ahokas and Salminen [48], binding efficiencies ranged widely, from 5.6% in *Lactococcus lactis* ssp. *cremoris* MK4 to 59.7% in *Lactobacillus amylovorus* CSCC 5160. Even within the same species, significant variation was observed: *Lactobacillus rhamnosus* strain Lc 1/3 bound 54.6% of AFB_1_, while strain E-97800 bound only 22.7%. These differences suggest that factors beyond peptidoglycan, such as cell surface components, contribute to binding efficacy. Binding is believed to occur via weak, noncovalent interactions involving hydrophobic pockets, as well as through glycopolymers such as teichoic acids embedded in the cell wall [5,48,70,71,72,74,75]. Teichoic acids, in particular, influence adsorption efficiency under varying pH conditions [5]. In addition, extrinsic parameters such as probiotic cell density, initial AF concentration, and temperature play a crucial role in determining the binding efficiency of probiotic strains [76]. Given the variability in AFB_1_-binding affinity across species and strains, certain LAB strains have been studied in greater detail for their probiotic value and detoxification potential. Among them, *Lactobacillus rhamnosus* GG and LC705 demonstrated particularly strong and stable binding to AFB_1_ across 12 LAB strains tested (*p* < 0.05). Moreover, heat and acid treatments further enhanced their binding capacity. Even after washing steps, viable *L. rhamnosus* GG and LC705 retained 50% and 38% of their bound AFB_1_, respectively [71]. Similarly, *Lactobacillus casei* L30 exhibited high binding affinity and stability among eight *L. casei* strains, maintaining AFB_1_ binding after washing and exposure to bile salts. Interestingly, the presence of bile salts increased the proportion of AFB_1_–*L. casei* complexes, suggesting that modifications to the bacterial cell envelope may improve binding interactions with AFB_1_ [75]. Due to their superior binding efficiency, strains such as *L. casei* and *L. rhamnosus* have been widely investigated for their potential role in preventing AFB_1_-induced hepatocarcinogenesis.

## 4. Anticarcinogenic Effect of Probiotics LAB on AFB_1_-Induced Liver Carcinogenesis

### 4.1. Probiotic Lactobacillus casei Shirota (Lcs)

**Figure 2:**
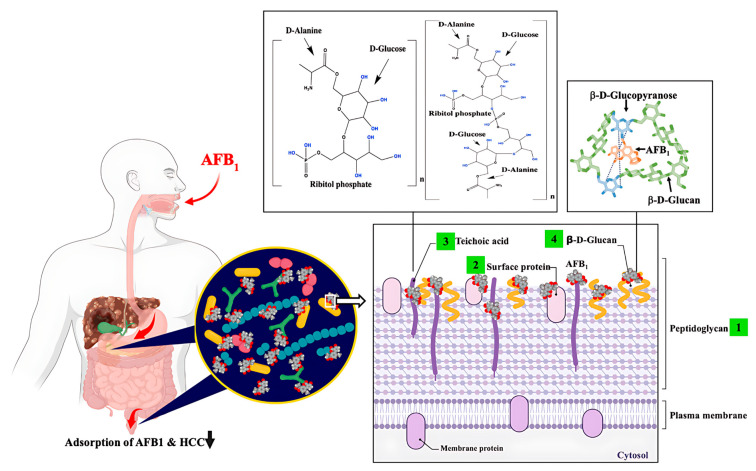
Proposed interactions between AFB_1_ and probiotic cell wall components in the duodenum of the small intestine. This schematic (not to scale) illustrates potential mechanisms by which probiotics reduce AFB_1_ adsorption and thereby lower the risk of HCC. AFB_1_ can interact with four major bacterial cell wall constituents: (1) peptidoglycans, (2) surface proteins, (3) wall teichoic acids, and (4) β-D-glucans. Variations in teichoic acid structures are represented for *Lactobacillus casei* Shirota and *Lactobacillus rhamnosus* GG as proposed by [83]. The suggested mechanism involves hydrogen bonding between hydroxyl groups of ribitol phosphate or glucose residues in teichoic acids and carbonyl oxygens of AFB_1_. Although these interactions are supported by experimental observations, the precise molecular mechanisms remain to be fully elucidated. AFB_1_–LAB complexes have been detected in both the duodenum and feces, supporting the concept that binding to probiotic surfaces inhibits intestinal absorption and reduces the likelihood of AFB_1_-induced carcinogenesis. Figure created with BioRender.com; chemical structures were prepared using ChemDraw v18.0.0.231.

The potential of probiotic LAB as a dietary strategy to mitigate HCC risk associated with AFB_1_ exposure has been demonstrated in both animal studies and human clinical trials (see Table 2). Probiotic *Lactobacillus casei* Shirota (Lcs) has been comprehensively studied due to its outstanding efficacy in detoxifying AFB_1_. Supplementation with Lcs significantly lowered systemic AFB_1_ levels in contaminated feed models and improved liver function biomarkers, such as alanine transaminase (ALT) and aspartate transaminase (AST), which are typically elevated during aflatoxicosis [77,78]. For example, ALT and AST levels rose to 108 U/L and 124 U/L, respectively, in AFB_1_-exposed animals without probiotics, but decreased to 75 U/L and 100 U/L in the Lcs-fed group [77]. Another study demonstrated that the Lcs treatment reduced AFB_1_ in the blood from 88 ng/mL to 50 ng/mL (*p* < 0.05) in AFB_1_-exposed rats [79]. These hepatoprotective effects were further supported by reductions in lipid peroxidation and improvements in histological outcomes [80]. In parallel, Lcs enhanced the activities of key antioxidant enzymes, including glutathione peroxidase (GPx), glutathione-S-transferase (GST), superoxide dismutase (SOD), and catalase (CAT), which counteract AFB_1_-induced oxidative stress [80,81,82]. Notably, GST facilitates detoxification by conjugating the reactive AFB_1_-8,9-epoxide intermediate with glutathione, producing the excretable metabolite AFB_1_-mercapturic acid in urine [81]. Together, these findings underscore the dual role of probiotics in lowering systemic toxin burden and reinforcing antioxidant defenses to preserve hepatic integrity [80,82]. Beyond enzymatic activity, bacterial cell wall structures contribute to AFB_1_ detoxification (see Figure 2). Serrano-Niño et al. [83] reported that teichoic acids, which constitute ~30% of the Lcs cell wall, undergo conformational changes upon AFB_1_ binding, as visualized by scanning electron microscope (SEM) [79]. The teichoic acid backbone, which is composed of glycerol and ribitol linked by phosphodiester bonds and decorated with glucose and D-alanine, provides hydroxyl groups capable of hydrogen bonding with AFB_1_ carbonyls (Figure 2) [83]. Strains deficient in teichoic acids exhibited markedly lower AFB_1_-binding efficiency, showing their importance in the detoxification process [5]. Other cell wall constituents also contribute; for example, β-D-glucans can capture AFB_1_ via hydrogen bonding and van der Waals interactions, particularly at the C(6)-hydroxyl group of glucopyranose residues [84]. Surface proteins likewise serve as binding sites, with heat-denatured Lcs showing enhanced AFB_1_ binding to unfolded proteins [79,85,86]. Collectively, these mechanisms demonstrate that AFB_1_-binding capacity is strongly strain-dependent and mediated by multiple structural components of the Lcs cell wall.

Building on animal studies, several clinical trials have assessed the efficacy of Lcs in reducing AFB_1_ absorption in humans. A prospective, randomized clinical trial recruited 71 healthy university employees with urinary aflatoxin M_1_ (AFM_1_) levels above 0.005 ng/mL, exploring whether fermented milk containing Lactobacillus casei probiotics could prevent AFB1 absorption in the GI tract [87]. According to the study, the concentrations of AFB1-lys in blood serum were reduced from 6.24 pg/mg to 5.48 pg/mg (*p* = 0.035) during the 4-week intervention period. Furthermore, a significant difference of 13.7% was observed between the placebo drink and milk with Lcs, at 6.35 pg/mg and 5.48 pg/mg, respectively, after 4 weeks of intervention (*p* = 0.005) [87]. Later, this research group recruited a broader range of participants (*n* = 174) from Selangor, Malaysia. After 12 weeks of intervention, the Lcs probiotics treatment group has a 23% reduction in AFM_1_ in excreted urine as compared to the placebo group [88]. These results provide compelling evidence that Lcs can be used as a dietary intervention to reduce AFB_1_ absorption and thereby lower the risk of long-term AF exposure in humans.

### 4.2. Probiotic Lactobacillus rhamnosus

Studies have also demonstrated that *L. rhamnosus* strains can substantially reduce the gastrointestinal absorption of AFB_1_. In chickens, duodenal uptake of AFB_1_ decreased by over 70% with *L. rhamnosus* GG (LGG) and by 37% with *L. rhamnosus* LC705, indicating that LGG exhibits stronger binding capacity than LC705 despite both belonging to the same species [89]. Similar findings were reported in rats: fecal excretion of AFB_1_ increased significantly within 24 h of LGG administration, and ALT activity was reduced, suggesting mitigation of AFB_1_-induced hepatotoxicity [90]. Subsequent experiments confirmed that LGG promoted the excretion of AFB_1_ in feces by forming stable LGG–AFB_1_ complexes within the GI tract, thereby reducing systemic toxicity [91]. As with other LAB strains (Section 4.1), the protective action of *L. rhamnosus* is also largely dependent on cell wall-mediated binding of AFB_1_ within the GI tract. Beyond adsorption, *L. rhamnosus* exhibits a distinct anti-inflammatory mechanism: it suppresses NF-κB signaling in AFB_1_-exposed liver tissue, thereby reducing the expression of proinflammatory cytokines (IL-1β, TNF-α, IL-6) and mitigating hepatotoxic responses [92].

The protective effects of *L. rhamnosus* have also been evaluated in clinical settings. In a randomized trial, El-Nezami et al. [93] showed that supplementation with *L. rhamnosus* LC705 significantly reduced urinary excretion of the DNA adduct AFB-N^7^-guanine. Levels decreased from 0.42 ng/mL to 0.27 ng/mL after 3 weeks (36% reduction) and to 0.19 ng/mL after 5 weeks (55% reduction) compared with placebo (*p* < 0.05). However, urinary AFB-N^7^-guanine levels returned to baseline after the intervention period, indicating that sustained probiotic intake may be necessary to maintain the detoxification effect [93]. All things considered, *L. rhamnosus* has an anti-inflammatory role and protective effects that attenuate the proinflammatory effects caused by AFB_1_ [90,92]. While clinical studies consistently show the potential of probiotics to reduce AFB_1_ absorption and related biomarkers, several limitations must be acknowledged. Many trials are restricted by relatively small sample sizes, short intervention durations (weeks rather than months), and variability in measured endpoints (e.g., urinary metabolites vs. serum adducts). These factors may limit generalizability and long-term risk assessment. Future research should focus on larger, multi-center trials with standardized protocols and extended follow-up to strengthen clinical evidence.

### 4.3. Mixture of Probiotic LABs

In addition to individual strains, mixtures of probiotic LABs have been investigated for their synergistic protective effects against AFB_1_-induced HCC. One study examined fermented milk containing a combination of *L. rhamnosus* GG (LGG) and *L. casei* Shirota (Lcs) in a 1:2 ratio. After 25 weeks of supplementation, this probiotic mixture markedly reduced both tumor incidence and tumor size in AFB_1_-exposed animals. At the molecular level, expression of oncogenes and proliferation-related factors including *c-myc*, *bcl-2*, *cyclin D1*, and *ras p21* was significantly downregulated. Since these genes are central to tumor progression and ROS-mediated pathways, the findings highlight the anti-hepatocarcinogenic potential of LGG–Lcs co-administration [94]. Another investigation evaluated a broader probiotic mixture composed of *L. reuteri*, *L. plantarum*, *L. pentosus*, *L. rhamnosus*, and *L. paracasei*. Chickens supplemented with this formulation exhibited a significant reduction in AFB_1_-induced liver enlargement, measured as relative liver weight (% EBW). Moreover, dietary supplementation decreased AFB_1_ accumulation in liver tissue by ~58% in the low-dose group (1000 ppb) and ~50% in the high-dose group (5000 ppb) (*p* < 0.05). Consistently, excretion of AFB_1_ in feces increased by 67% and 46% at the respective dose levels compared to unsupplemented controls [95]. Mixtures of probiotics may offer a more practical dietary approach than single strains, reflecting the diversity of probiotics naturally present in fermented foods. By combining different binding capacities, enzymatic defenses, and immunomodulatory functions, such formulations could provide broader protection against AFB_1_ and hold promise as feasible interventions for populations with regular dietary exposure.

**Table 2:** In vivo experiments: Anticarcinogenic effect of probiotic LABs on AFB_1_-induced liver carcinogenesis.

Subjects	Dose of AFB_1_	Treatment Period	Anti-Hepatocarcinogenic Functions	LAB Strains	Ref
Male Sprague Dawley rats (7–8 weeks old)	25 ppb	Daily for 20 days	ALT & AST ↓ Serum AFB_1_ ↓	*L. casei* Shirota	[77]
Male Sprague Dawley rats (7–8 weeks old)	25 ppb	Daily for 5 days	Serum AFB_1_ ↓	*L. casei* Shirota	[79]
Male Wistar rats (4 weeks old)	450 ppb	Twice/week for 6 weeks	TBARS ↓ Antioxidant enzymes ↑	*L. casei* Shirota *L. rhamnosus* GG	[80]
71 employees in UPM	Urinary AFM_1_ > 0.005 ppb	4 weeks of intervention	Serum AFB_1_ ↓	*L. casei* Shirota	[87]
Broiler chickens (1 week old)	3000 ppb	Single injection	AFB_1_ in duodenal tissue & luminal fluid ↓	*L. rhamnosus LC705* *L. rhamnosus GG*	[89]
Han-Wistar rats (5 weeks old)	1500 ppb	Daily for 3 days	ALT ↓ AFB_1_ in feces ↑	*L. rhamnosus GG*	[90]
Male Holstein calves (120 days old)	38 ppb	Single oral	AFB_1_ in feces ↑	*L. rhamnosus GG*	[91]
Male Kunming mice (5 weeks old)	300 ppb	Twice/day for 8 weeks	Inflammatory factors ↓ ALT & AST ↓	*L. rhamnosus*	[92]
90 male students at Sun Yat-Sen University	Urinary AFM_1_ > 0.008 ppb	Twice/day for 5 weeks of intervention	Urinary AFB-N^7^-guanine ↓	*L. rhamnosus LC705*	[93]
Male Wistar rats (4 weeks old)	450 ppb	Twice/week for 25 weeks	*c-myc, bcl-2, cyclin D1 & rasp-21* ↓ Tumor incidence ↓	Mixture of *L. casei Shirota &* *L. rhamnosus GG*	[94]
Male Ross broiler chicks (1 day old)	Low (1000 ppb) High (5000 ppb)	Daily for 35 days	Liver EBW ↓ AFB_1_ in liver tissue ↓ AFB_1_ in excreta ↑	Mixture of LAB	[95]

ALT: alanine transaminase; AST: aspartate transaminase; EBW (empty body weight) = body weight before sacrifice—weight of alimentary tract filled with chime; ppb: part per billion (1 ppb = μg/kg), ↑: indicates increase, ↓: indicates decrease.

## 5. Conclusions

AFB_1_ remains a significant global challenge to food safety and public health, with chronic dietary exposure strongly associated with hepatocellular carcinoma (HCC). Probiotic lactic acid bacteria (LAB) have emerged as promising biological countermeasures, supported by growing evidence from mechanistic studies, animal experiments, and human clinical trials. These probiotics, including *Lactobacillus casei* Shirota, *Lactobacillus rhamnosus* GG, *Lactobacillus rhamnosus* LC705, *Lactococcus lactis*, and selected *Bifidobacterium* species, mitigate AFB_1_ toxicity through multiple complementary mechanisms. The most compelling evidence comes from clinical intervention trials, which consistently demonstrate reductions in urinary AFM_1_ and DNA adducts following probiotic supplementation, highlighting clear translational potential. Moreover, findings from mechanistic and animal studies—such as enhanced antioxidant activity, NF-κB pathway modulation, intestinal barrier restoration, and shifts in gut microbiota—provide valuable insight but still require confirmation in long-term human populations. Looking ahead, approaches such as engineered probiotics, probiotic–prebiotic combinations, and standardized multi-strain formulations represent exciting future directions, though they remain largely hypothetical and demand rigorous evaluation. At the molecular level, probiotic protection is largely mediated by cell wall components such as peptidoglycans, teichoic acids, β-D-glucans, and surface proteins, which adsorb AFB_1_ and limit its intestinal absorption. These interactions emphasize the importance of strain selection and mechanistic characterization, particularly given the variability in binding efficiency across strains. To advance translation, further biochemical studies are needed to clarify structural mechanisms, and large-scale randomized controlled trials in high-risk populations are required to establish efficacy, optimize dosing regimens, and evaluate the synergistic effects of probiotic mixtures. From a practical perspective, probiotics are generally safe, affordable, and widely accepted within dietary contexts, making them attractive candidates for large-scale food safety interventions. Nonetheless, regulatory approval, product standardization, and quality control can remain critical challenges to their widespread application. In summary, probiotic LABs represent safe, cost-effective, and scalable interventions for reducing AFB_1_ exposure and preventing HCC. Their multifaceted mechanisms of action highlight their promise as a cornerstone of global mycotoxin management, provided that ongoing mechanistic research and clinical validation bridge the gap between laboratory potential and real-world implementation.

## Figures and Tables

**Table 1:** In vivo experiments: Gut-health induced microbiota alteration caused by AFB_1_.

Subjects	Dose of AFB_1_	Treatment Period	Microbial Community Alteration	Ref
Dorper Mutton Sheep	1000 ppb (1/2 LD50)	One time	*Firmicutes* ↑ *Spirochaetes* ↑ *Proteobacteria* ↑ *Actinobacteria* ↓ *Bacteroidetes* ↓	[43]
Male Balb/c mice	25 ppb (1/192 LD50)	Daily for 28 days	*Parabacteroides* ↑ *Escherichia-Shigella* ↑ *Lactobacillus* ↑ *Alistipes* ↓ *Bacteroidetes* ↓	[44]
Dorper Mutton Sheep	1000 ppb (1/2 LD50)	One time	*Firmicutes* ↑ *Spirochaetes* ↑ *Verrucomicrobia* ↑ *Proteobacteria* ↑ *Bacteroidetes* ↓	[45]
Male Balb/c mice	25 ppb (1/192 LD50)	Daily for 28 days	*Firmicutes* ↑ *Lactobacillus* ↑	[46]
Male Fischer 344 rats (5 weeks old)	Low (5 ppb) Medium (25 ppb) High (75 ppb)	5 days/week for 4 weeks	*Streptococcus* spp. & *Lactococcus* spp. ↓	[49]
Crossbred TOPIGS-40 hybrid piglets	320 ppb	Daily for 30 days	*Lactobacillus* ↓	[50]
Male broiler chicks (1-day old)	Low (1000 ppb) Medium (1500 ppb) High (2000 ppb)	Daily for 21 days	Total LAB ↓ with low AFB_1_ Total LAB ↑ with medium and high dosage of AFB_1_	[51]
Kunming mice	Low (2500 ppb) Medium (4000 ppb) High (10,000 ppb)	Twice/day for 62 days	*Bifidobacterium* spp. ↑	[53]

LD50: median lethal dose, ppb: part per billion (1 ppb = μg/kg), ↑: indicates increase, ↓: indicates decrease.

## Data Availability

No new data were created or analyzed in this study.
